# Modelling the impact on a local mental health system of previously implemented care programs: the experience of assertive outreach teams in Bizkaia (Spain)

**DOI:** 10.1017/S2045796025000125

**Published:** 2025-03-17

**Authors:** N. Almeda, D. Diaz-Milanes, H. Killaspy, L. Salvador-Carulla, J. J. Uriarte-Uriarte, C. R. García Alonso

**Affiliations:** 1 Department of Psychology, Universidad Loyola Andalucía, Seville, Spain; 2 Department of Quantitative Methods, Universidad Loyola Andalucía, Seville, Spain; 3 Institute of Health Research, University of Canberra, Canberra, Australia; 4 Faculty of Brain Sciences, Division of Psychiatry, University College London, London, UK; 5 Bizkaia Mental Health Services, Osakidetza-Basque Health Service, Biocruces Health Research Institute, Bilbao, Spain

**Keywords:** assertive community treatment, assertive outreach, causal modelling, ecosystem, relative technical efficiency, research implementation

## Abstract

**Aims:**

The study assessed the interactions and the impact of specialist mobile community care teams (assertive outreach teams or AOTs) implemented in the mental health (MH) system of Bizkaia (Spain) using a methodology derived from an ecosystem perspective.

**Methods:**

First, the experts assessed the system’s services and codified them according to an international classification system. Second, following an iterative methodology for expert-knowledge elicitation, a clients’ flow diagram showing the inter-dependencies of the system’s components was developed. It included variables and their relationships represented in a causal model. Third, the system elements where the AOTs had a major impact (stress nodes) were identified. Fourth, three scenarios (variable combinations representing the ‘stress points’ of the system) were modelled to assess its relative technical efficiency (technical performance indicator).

**Results:**

The classification system identified the lack of fidelity of the AOTs to the original assertive community treatment model, categorizing them as non-acute low-intensity mobile care. The causal model identified the following elements of the system as ‘stress nodes’ in relation to AOT: users’ families; social services (outside of the healthcare system); acute hospitals; non-acute residential facilities and, to a lesser extent, acute hospital day care services. When the stress nodes inside the healthcare system were modelled separately, acute and non-acute hospital care services resulted in a large deterioration in the system performance, while acute day hospital care had only a small impact.

**Conclusions:**

The development of the expert-knowledge-based causal model from an ecosystem perspective was helpful in combining information from different levels, from nano to macro, to identify the components in the system likely to be most affected by a potential policy intervention, such as the closure of AOTs. It was also able to illustrate the interaction between the MH system components over time and the impact of the potential changes on the technical performance of the system. Such approaches have potential future application in assisting with service planning and decision-making in other health systems and socio-economic contexts.

## Introduction

The deinstitutionalization process in recent decades has aims to transform mental health (MH) systems from hospital-based to community-based (Thornicroft *et al.*, 2016). However, the MH systems in the European Union has faced several challenges and transformations, resulting in this shift being implemented unevenly across its countries and regions (Vandoni *et al.*, 2024). This disparity has led to the development of a wide variety of complex MH structures with high-dimensional interactions that are challenging to model using classical methods (Salvador-Carulla *et al.*, 2013b). Additionally, the literature lacks practical guidance on implementing complex interventions in real-world settings (Datta and Petticrew, 2013).

From an Evidence-Based Medicine point of view, implementation of specific programs or services within a MH system has often been decided on the basis of randomized control trials. Although RCTs have many benefits, particularly in assessing the efficacy of simple interventions for acute conditions, they are not as useful in the context of multimorbidity and chronicity that tend to characterize clients of complex healthcare systems (Fernandez *et al.*, 2015). A good example is the contradictory results about the effectiveness and efficiency of assertive community treatment (ACT) teams, whose effectiveness has been shown to vary according to the degree to which fidelity to the original model is adhered to, overlap with other existing services and context specific variance in key system components, such as inpatient service availability and threshold for hospital admission (Burns, 2010).

The ACT model comprises an intensive community-based approach, delivered in people’s homes by a multidisciplinary MH team to people with severe mental illness who are high users of inpatient psychiatric care. It was originally developed in the US and Australia to provide an alternative approach to people experiencing repeated cycles of relapse and readmission with the aim of minimizing both (Hoult, 1986; Stein and Test, 1980). The original ACT model described a 24-hour service, staff with a low caseload (client:staff ratio 1:12) and long-term clients (Bond and Drake, 2015). However, as the model spread globally, variation in the degree to which the original model was implemented were identified, with few teams being considered as ‘high fidelity’ (Wright *et al.*, 2003). This fact, plus the lack of a general framework that justifies the choice of analysis units, could compromise the replicability and comparability of the studies (Furst *et al.*, 2019).

Furthermore, even in high model fidelity teams, assessment of the success of local implementation may be questionable unless the local context is considered (Raine *et al.*, 2016; Salvador-Carulla *et al.*, 2017). Although it is well known that the effect of an intervention depends on the characteristics of the local context (Furst *et al.*, 2021), this is rarely studied (Raine *et al.*, 2016). The inclusion of more detailed information on the intervention, context and outcomes in quantitative studies (Datta and Petticrew, 2013) could reduce the gap between research and practice (Salvador-Carulla *et al.*, 2013b).

Despite this, particularly in Europe, low-fidelity versions of ACT are being implemented without comprehensive evaluation (Rosen *et al.*, 2013). As a result, many MH systems have struggled to provide adequate care due to two main factors: (a) competing demands on the overall health and social care system, and (b) insufficient coordination and understanding of the broader impacts on other sectors, such as social care and the justice system (Rosen *et al.*, 2020). Furthermore, assessment of the impact of new services often relies on high level data such as national admission rates, which is uninterpretable at the local level (Rosenberg *et al.*, 2015) and can result in a lack of accountability and ecological fallacy (Furst *et al.*, 2019; Rosen *et al.*, 2020).

In Spain, MH systems have shifted from deinstitutionalization to a person-centred approach based on the balanced care model (Thornicroft and Tansella, 2013) and the recovery model (Slade *et al.*, 2014). The Spanish Mental Health Strategy 2022–2026 encourages individuals with MH issues to take an active role in their recovery process (Suárez Alonso *et al.*, 2022). Nonetheless, significant inconsistencies in service delivery persist across regions. For instance, as of 2010, psychiatric hospitals remained operational in some areas, such as the Basque Country, while other regions like Andalusia had fully closed them (Salvador-Carulla *et al.*, 2010). The lack of a standardized system for service provision complicates comparative analysis of financing and effectiveness (Salvador-Carulla *et al.*, 2020).

A framework considering insights from implementation research, particularly context and impact analysis, is needed to address these inconsistencies (Salvador-Carulla *et al.*, 2006, 2017). The ecosystem approach integrates the complex dynamics of MH care systems, aiding in the analysis of intervention impacts and informing policy and practice (Furst *et al.*, 2021).

This study aimed to analyse, using an ecosystem approach, the relevance of assertive outreach teams (AOTs), a local adaptation of ACT, on the technical performance of a Spanish MH system (Basque Country).

## Methods

### Setting

The MH Network of Bizkaia was selected as the study area. Bizkaia is one of the three provinces of the Basque Country autonomous community (Spain), which has implemented a centralized MH network (adult population 1,137,000 inhabitants, 2015). This system integrates hospital and community services in 19 catchment areas, each with a reference community MH centre. In addition, it has three general hospitals for acute care, four centres for children and adolescent MH care, fifteen day care hospitals, one educational therapeutic centre, five AOTs (Comarca Interior, Ezkerraldea, Uribe, Homeless Bilbao and Bilbao) and one day care hospital for addictions (Gutiérrez-Colosía *et al.*, 2021). In 2015, the AOTs provided care to 317 clients plus 78 clients assigned to Homeless Bilbao (See Supplementary Material 1 for details). The Homeless Bilbao AOT’s does not specifically aim to avoid hospital admissions; for that reason, this service was excluded from this study.

### Services classification and causal model design

All the services were classified according to their main types of care using the Description and Evaluation of Services and DirectoriEs for Long Term Care (DESDE-LTC) (Salvador-Carulla *et al.*, 2013a) and the corresponding glossary of terms (Gutierrez-Colosia *et al.*, 2022; Montagni *et al.*, 2018).

The Expert-based Cooperative Analysis (EbCA) (Gibert *et al.*, 2010) was conducted to identify the interactions between AOTs and other types of care, social and voluntary care and family support. Senior managers, psychiatrists, psychologists, nurses and experts in service planning and management participated in the meetings, and their knowledge was formalized to describe how clients ‘flow’ through the MH system, as well as to identify the variables used to assess technical performance.

### Design of scenarios

The multidimensional nature of the Bizkaia MH system was studied carefully to determine the ecosystem components in the clients’ flow and the ‘stress’ nodes (i.e. critical ones) were identified by the experts (EbCA). Sets of interrelated variables (called scenarios) were designed by the experts for assessing technical performance.

Variables were organized into inputs and outputs. Inputs refer to the resources used by MH services (e.g. placement capacity, workforce capacity, etc.) to produce outputs (e.g. length of stay [LOS], discharges, readmissions, prevalence, etc.).

### Data processing and analysis

The codesigned scenarios were analysed by the EDeS-MH decision support system (García-Alonso *et al.*, 2019b). According to the formalized expert knowledge, all the variables were transformed into statistical distributions, and their parameters were selected to represent the with- (original) and without-AOTs (after a theoretical AOT removal) situations. A Monte-Carlo simulation engine assessed the randomness and resulting uncertainty of the system.

Simulated variable values were interpreted in terms of ‘appropriateness’ according to the Balanced Care Model (Thornicroft and Tansella, 2013, 2004) paradigm. Knowledge was formalized by standard IF … THEN … rules. A fuzzy inference engine stablishes the rules activating the corresponding interpretation (linear monotone increasing/decreasing functions).

Relative technical efficiency (RTE) was selected as the indicator to assess the MH ecosystem technical performance. Data envelopment analysis with variable returns to scale and input orientation was selected (Banker *et al.*, 1984). Additionally, ecosystem stability and Shannon’s entropy were also assessed. Stability measures the sensitivity of RTE scores to changes in input/output values. This indicator is assessed in a 0 (completely unstable) to 100 (completely stable) scale. A system can be considered stable when RTE does not vary (or has minimum changes) in response to input/output variations (usually due to structural changes in services or types of care). On the other hand, if RTE scores vary a lot when inputs/outputs suffer significant changes then the system under evaluation is considered unstable. Shannon’s entropy is the selected indicator to assess if the system management can be considered (minimum entropy) or not (maximum entropy) homogeneous. Person-centred MH systems use to have higher entropy values than those based on institutional services (Almeda *et al.*, 2022a; García-Alonso *et al.*, 2019b).

### Simulation process

Asymmetric triangular distributions were selected for each variable, with the original values as the modes, and minima/maxima set at −5% and +10% of the modes. Each scenario involved 1000 simulation runs.

## Results

### AOT clients’ flows in the ecosystem and the potential effect of removal of AOTs

**Figure 1.**
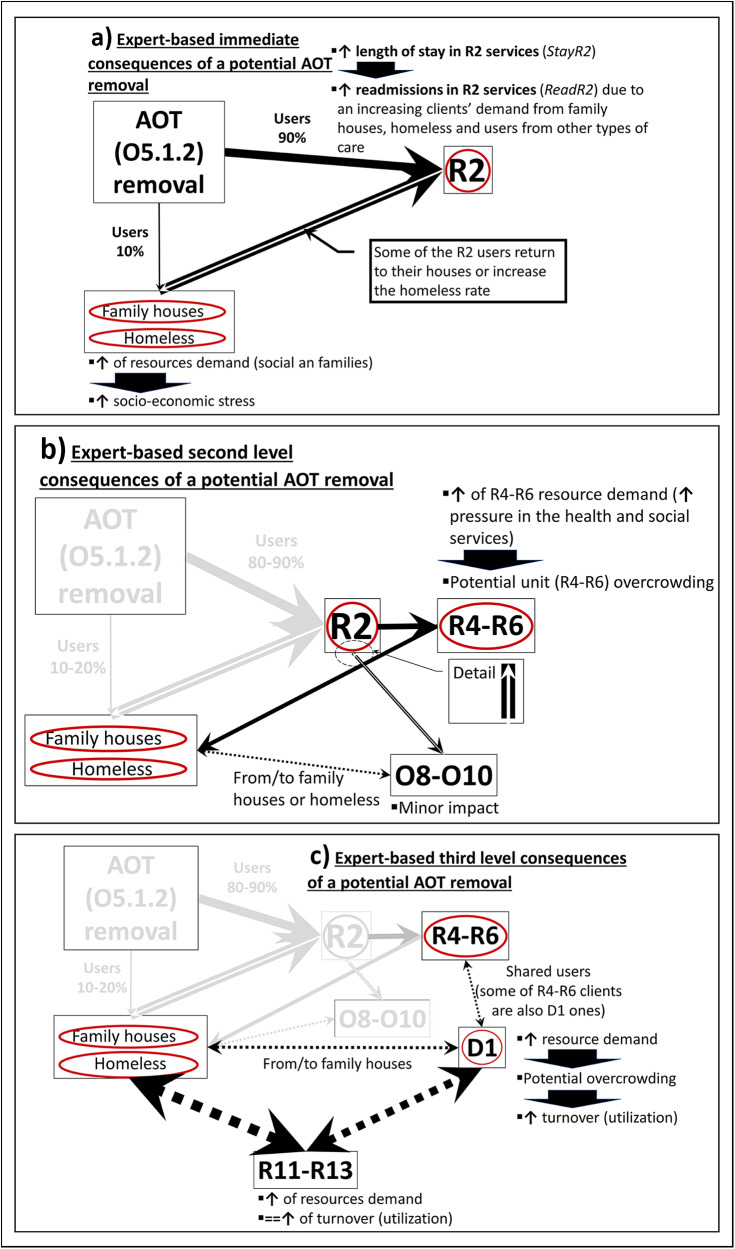
Users’ flowchart showing the relationships and consequences after a potential removal of ACT teams (from EbCA process). Identified main stress points were highlighted by red circles.

Based on EbCA, experts identified the ecosystem components directly related to AOTs and described the nature of their relationships. AOTs (O5.1.2 DESDE-LTC code) were explicitly designed to decrease readmissions (ReadR2) and LOS (StayR2) in acute hospital care in Bizkaia (R2 DESDE-LTC code). Any change in their structure would be expected to have an immediate negative impact (Fig. 1a).

**Figure 2.**
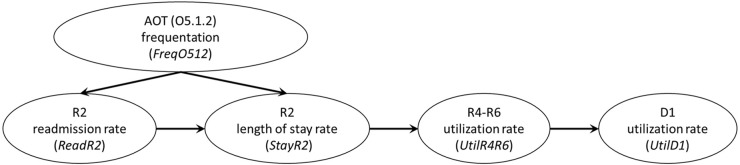
Expert-based potential causal consequences of AOTs removal.

According to the experts’ knowledge, potential removal of the AOTs without some compensatory measures would cause acute hospital care (R2) overload (StayR2 and ReadR2 would increase). Trying to solve this situation, decision-makers in R2 services would increase early discharges rates (trying to maintain StayR2 stable), which may affect (1) the families of the more autonomous clients by increasing their socio-economic stress (social effect) as they would be likely to provide support to their relatives, (2) the non-acute time-limited hospital care (DESDE-LTC codes R4–R6) services (potential overcrowding) for the less autonomous ones and, in to a lesser degree, (3) non-acute outpatient care (DESDE-LTC codes O8-O10) (Fig. 1b). Here the number of services and professionals could not be modified (AvR4R6) and the negative effects were focused on the rates of utilization (UtilR4R6 and UtilD1, Fig. 2).

At this point, some of the less autonomous AOT clients may become homeless due to their engagement difficulties and the potential lack of beds in non-acute time-limited hospital care (R4–R6) services. This second potential effect would increase these individuals’ readmission rates in acute hospital care (R2) and/or socio-economic stress to their families. Consequently, this process may accelerate the users’ turnover in non-acute time-limited hospital care (R4–R6) trying to meet population needs. On the other hand, the users with family and social support would potentially continue their outpatient follow-up care visits (08–O10) or, alternatively, the majority could be referred to acute hospital care (R2).

A fraction of users living with their families or in non-acute time-limited hospital care services (R4–R6) would become new users of acute day hospital care services (DESDE-LTC code D1), increasing their overload and provoking potentially greater turnover (Fig. 1c).

Finally, the users who develop good self-efficacy and independency in health and daily activities management could be transferred to non-hospital residential care services (DESDE-LTC codes R11–R13), maintaining their follow-up in outpatient care (O8–O10) and/or their places in acute day hospital care (D1) services (Fig. 1c).

Due to the clients’ flow and the identified interrelationships between the system components, the experts concluded that if the AOTs were removed, the stress nodes would be the clients’ families and social services outside of the healthcare system, acute hospitals (R2), non-acute time-limited hospitals (R4–R6) and, to a lesser extent, acute hospital day (D1) care services.

### Scenario design and impact estimation

Considering the stress nodes, three scenarios were designed (considering the healthcare system): (S1) acute hospital (R2), (S2) non-acute time-limited hospital care (R4–R6) and, finally, (S3) acute day care (D1) (Table 1).

**Table 1.** Scenarios and variables directly affected by the potential removal of AOT

Scenarios	Inputs	Outputs
**S1: Acute hospital care (R2)**	1. Availability of R2 facilities (*AvR2*), rate per 100,000 adults	1. Readmissions in R2 (*ReadR2*), number of readmissions/number of discharges rate per 1,000 adults
	2. Number of beds in R2 (*BedR2*), rate per 100,000 adults	2. Length of stay in R2 (*StayR2*), number of days in R2/total admissions rate per 1,000 adults
	3. Workforce capacity: Nº of psychiatrists (*PsychiatR2*), psychologists (*PsycholR2*), nurses (*NurR2*) and the total of professionals (*ProfR2*) in R2, all rates per 100,000 adults	
**S2: Non-acute hospital care (R4–R6)**	1. Availability of R4–R6 facilities (*AvR4R6*), rate per 100,000 adults	1. Utilization in R4–R6 facilities (*UtilR4R6*), places and beds in 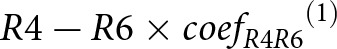 rate per 1,000 adults
	2. Number of beds in R4–R6 (*BedR4R6*), rate per 100,000 adults	
	3. Workforce capacity: Nº of psychiatrists (*PsychiatR4R6*), psychologists (*PsycholR4R6*), nurses (*NurR4R6*) and the total of professionals in R4–R6 (*ProfR4R6*), all rates per 100,000 adults	
**S3: Acute day hospital care (D1)**	1. Availability of D1 facilities (*AvD1*), rate per 100,000 adults	1. Utilization in D1 facilities (*UtilD1*), places in 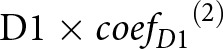 rate per 1,000 adults
	2. Number of places in D1 (*PlaD1*), rate per 100,000 adults	
	3. Workforce capacity: Nº of psychiatrists (*PsychiatD1*), nurses (*NurD1*) and the total of professionals in D1 (*ProfD1*), all rates per 100,000 adults	

*Notes*: (1) Taking into consideration the real situation of the ecosystem 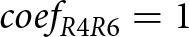, (2) Taking into consideration the real situation of the ecosystem 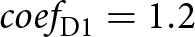.

The clients’ flow (Fig. 1) was used to determinate the corresponding value modifications on the output variables considering two situations. First, the experts thought that a relevant number of AOT clients could have enough social support to return home or be easily transferred to residential facilities (‘low-demand of care’ situation). Second, the experts considered that the needs of AOTs’ users could be too complex and, consequently, the MH ecosystem would transfer them quickly to adequately supported settings (‘high-demand of care’ situation). This process is described in Table 2.

**Table 2.** Analysing the impact of AOT: modifications in original data values according to the Bayesian network

Both situations	
According to expert knowledge, if the AOTs (O5.1.2) were removed from the MH system without any compensatory measure, acute hospital care services (R2) must firstly be ready to provide care up to ≈90% (the uncertainty here is very high^(1)^) of the 239 AOT clients because these services mediated the clients’ access to: non-acute time-limited hospital (R4–R6) and day hospital (D1) services (Fig. 1a, b).	
Considering the structure of the analysed MH system, from R2 services, most of the clients (at least ≈50%) would be transferred to R4–R6 facilities (Fig. 1b), because of the high probability of readmission in R2 units, while a few of them with enough independence and social support would be referred to a non-acute outpatient (O8–O10) service for follow-up (Fig. 1b).	
Following the organization of the MH system, up to ≈30% of the original AOT clients could have an adequate condition to be transferred to a day care service (D1, Fig. 1b) while they live with their family or in a R11–R13 units (Fig. 1c). Finally, a group of clients would be shared by R4–R6 and D1 units as a part of their rehabilitation process preparing them for a potential discharge to the community in other to make free the bed (Fig. 1c).	
The clients’ frequentation (*FreqO512*, number of visits or contacts in the AOTs) was used to increase or decrease the length of stay (*StayR2*) and the readmission (*ReadR2*) rates in acute hospital care as well as the utilization rate in residential non-hospital care (*UtilR4R6*) and, finally, the utilization rate in day care (*UtilD1*) (Fig. 2).	
Frequentation of AOTs (*FreqO512*) was a proxy for the medical condition, degree of independence, health status or social support of the AOT clients.	
**Low-demand situation**	**High-demand situation**
In acute hospital care (R2), the readmission rate (*ReadR2*) increases in a range of 2–3 times per year for each AOT client (up to ≈90% from the original AOT clients), being the corresponding length of stay rate (*StayR2*) on average in a range of 2–2.5 times greater than the actual one.	In acute hospital care (R2), an increase in a range of 5–7 times per year in the readmission rate (*ReadR2*) is expected for each AOT client (up to ≈90% from the original AOT clients). For their length of stay rate (*StayR2*) on average, an increase in a range of 2.5–4 times from the actual one, for each AOT client, is also expected.
In residential non-acute time-limited hospital care (R4–R6), all the corresponding AOT clients (up to ≈50% from the original ones) must be allocated (*UtilR4R6*), no matter their demand profiles (*FreqO512)*.	In residential non-hospital time-limited services (R4–R6), an increase of ≈2 times the utilization rate (*UtilR4R6*) on average is expected (up to ≈50% from the original ones). But it could increase up to ≈4 times depending to client’s demand profile (*FreqO512)*. Therefore, the discharge process from R2 units could be more and more difficult. This would happen if acute hospital care (R2) had to discharge patients quickly to reduce *StayR2*, but most of them do not have enough social support and independence to stay in a R11–R13 unit or living with their family and neither are suitable for day care (D1).
In the case of day care (D1), the utilization rate (*UtilD1*) should vary according to a coefficient in a range [0.95, 1.1] that multiplies the number of ≈30% of AOT clients. This range slightly modify the current utilization rate due to two potential factors: (1) if enough clients have good social support and independence, then they do not need a place in D1, and (2) if some of the clients have a worse health condition, then they could not be assigned to a day care facility (D1).	In the day care services (D1), utilization rate (*UtilD1*) should vary according to a coefficient in a range [1, 1.4] that multiplies the number of ≈30% of AOT clients, considering that they are suitable to be D1 clients but also are family dependent or they stay in a R11–R13 or R4–R6 unit.

*Note*: (1) Uncertainty is dealt by the Monte-Carlo simulation engine.

### Policy impact on service outputs

In scenario 1 (S1) and ‘low-demand of care’ situation, ReadR2 increased up to 10% on average (min: 5.56; max: 14) to 34.11% (min: 24.74; max: 50.82). On the other hand, StayR2 increased by 54.83% in the whole ecosystem, which meant an increase from 16 days (min: 11.05; max: 19.88) to almost 25 days (min: 15.38; max: 34.19) per user on average.

In S1 and ‘high demand of care’ situation, the ReadR2 increased up to 68.63% on average (min: 47.37; max: 108.2) and StayR2 reached 46 days on average (min: 25.67; max: 75.05).

In scenario 2 (S2) and ‘low demand of care’, UtilR4-R6 could increase from 0.33 (min: 0.28; max: 0.42) to 0.45 (min: 0.38; max: 0.53) places/1000 adults on average. In the ‘high demand of care’ situation, UtilR4R6 may increase up to 0.65 (min: 0.54; max: 0.73) on average.

Finally, in scenario 3 (S3) and ‘low demand of care’, UtilD1 on average could increase from 0.06 (min: 0.04; max: 0.07) to 0.13 (min: 0.1; max: 0.17) places/1000 adults. In ‘high demand of care’, UtilD1 could reach up to 0.14 (min: 0.10; max: 0.18) on average.

### Impact on acute hospital care (R2) services technical performance

See Supplementary Material 2 for more details about the RTE analysis.

In S1 and, respectively, for the ‘low demand of care’ and ‘high demand of care’ situations, results showed a reduction of −0.97% (from 0.913 to 0.904) and −1.47% (from 0.913 to 0.9) on the global RTE on average. The statistical error was always lower than 2.5%, and the probability of having an RTE greater than 0.75 remained almost constant.

Globally, in S1, the system’s stability decreased in both situations, up to −4.34% (from 60.34 to 57.73) and −5.14% (from 60.34 to 57.24) respectively. The entropy decreased up to −2.18% (from 42.59 to 41.66) and −2.04% (from 42.59 to 41.72), respectively.

### Impact on non-acute time-limited hospital care (R4–R6) services technical performance

In scenario 2 (S2), the global RTE on average decreased slightly by −0.26% in the ‘low demand of care’ situation (from 0.888 to 0.885) and strongly −1.04% in the ‘high demand of care’ one (from 0.888 to 0.878). The statistical error was always below 2.5%.

For the whole ecosystem, the probability of having an RTE greater than 0.75 remained almost constant (from 96.1% to 96.62%).

The global stability was almost constant (−0.62%) for the ‘low demand of care’ situation but in the ‘high demand of care’ one it decreased up to −4.93%. The entropy of the ecosystem remained almost constant in the ‘low demand of care’ situation, but it decreased up to −1.33% in the ‘high demand of care’ one.

### Impact on acute day hospital care (D1) services technical performance

In scenario 3 (S3), the RTE on average remained almost constant in both situations. For the ‘low demand of care’ one, the RTE on average decreased only −0.27%, while for the ‘high demand of care’ was −0.37%. The probability of having RTE greater than 0.75 also remained constant. The statistical error was always lower than 2.5%.

The stability of the global ecosystem increased up to 3.42% and 3.34%, respectively, for both situations. The global entropy increased up to 0.94% in the ‘low demand of care’ situation and up to 2.45% in the ‘high demand of care’ one.

## Discussion

The study examines the impact of a complex type of care, AOTs in the MH system of Bizkaia (Spain). To accomplish this objective, a causal model was designed by a group of experts (EbCA), which estimated the clients’ flow into the MH system and its context, the stress nodes (Bayesian network) generated if AOTs were removed, and their impact on MH system performance.

Regarding the classification, the AOTs considered initially by the experts as ACT teams did not follow closely the criteria of fidelity to the original model (Teague *et al.*, 1998); being a diluted ACT version (Wright *et al.*, 2003). All the AOTs were classified as low-intensity non-acute mobile care (O 5.1.2 DESDE-LCT code) and were the only ones that provided mobile care, so no evidence of overlapping with other services was found.

Studies of AOTs with higher fidelity have found them to be associated with a greater reduction in clients’ hospitalization (Marshall *et al.*, 1998), showing a decrease of 23% more than lower fidelity teams (Latimer, 1999). However, a meta-regression of multiple trials of AOTs showed that the level of fidelity had only a fairly limited effect on reducing hospital use (Burns, 2010; Burns *et al.*, 2007). In contrast, adapting the AOTs to the local context could be more valuable (Bond and Drake, 2015; Rosen and Salvador-Carulla, 2022).

The mapping and classification of the Bizkaia MH system allowed a robust, standardized description of the services available and an understanding of the capacity, workforce and function they provided (Rosen and Salvador-Carulla, 2022). This process allows identification of comparable units and can detect overlap in service functionalities.

Considering the causal model and the clients’ flow, the analysis endorsed the positive impact of the AOTs on the technical performance of the MH system of Bizkaia. The expert-based model (Figs. 1 and 2) indicated that, initially, the acute hospital care (R2) services would experience the direct impact of a potential removal of AOTs through increased readmissions and LOS which would increase the need of non-acute time-limited hospital care (R4–R6) services as well as healthcare costs (Pereira-Rodríguez *et al.*, 2012).

However, the integration of AOTs will not always lead to substantial reductions in hospital use (Burns *et al.*, 2007; Randall *et al.*, 2015). In the UK, the standard community MH teams obtained similar results to those obtained by high-fidelity ACT teams (Killaspy *et al.*, 2006, 2009), although, across the UK, many were not adequately staffed to deliver the AOT approach (Ghosh and Killaspy, 2010). In contrast, the MH system of Bizkaia is a complex community-based MH system strongly supported by AOTs. The potential removal of those teams would generate a significant increment in hospital use, worse community-based care and poorer performance (Burns *et al.*, 2007).

Without the AOT teams, the non-acute time-limited hospital care (R4–R6) services would become overcrowded, requiring an increase in the LOS in acute hospital care (R2) services, which would likely result in early discharges in order to prevent bed congestion. Under these circumstances, some of the less autonomous clients may become homeless (Penzenstadler *et al.*, 2019). This situation might lead to either a prolonged LOS or discharge with a high risk of readmission (Hwang and Burns, 2014). In these cases, transferring these users to other residential or hospital services would be key to maintain the continuity of care if AOTs are unavailable.

The experts indicated the most autonomous individuals could potentially obtain a place in acute day hospital care (D1) services. Without adequate attention, these individuals may become involved in criminal activity, struggle with drug abuse or suffer the negative effects of marginalization and self-neglect (Aagaard *et al.*, 2017; Drake and Latimer, 2012; Rosen *et al.*, 2013).

The ACT model has shown to be effective in reducing the number of visits to psychiatric emergency rooms and hospitalizations (Aagaard *et al.*, 2017). In this regard, the removal of the AOTs in Bizkaia should not significantly affect non-mobile outpatient care (O8–O10) services since their clients do not usually keep outpatient appointments. In addition, the non-hospital residential care (R11–R13) services only accept individuals with a high level of autonomy and adequate health management. For those reasons, removal of AOTs would have only a minimal impact on outpatient and non-hospital residential care services.

Regarding the development of the causal model itself, the process was able to produce a graphical description of the essential components within the MH system and their relationships (Anderson *et al.*, 2011; Hawe *et al.*, 2009). This process allows assessment of how a change will impact on other related sections of the system, allowing for better understanding of approaches to intervention and evaluation design (Hawe *et al.*, 2004). This method also allows impact assessment in the short and long term, thanks to the inclusion of expert knowledge (Datta and Petticrew, 2013). The further interpretation and analysis of the impact of changes to the system on the identified stress nodes, further enables the planning process to be more transparent and causal mechanisms more explicit (Anderson *et al.*, 2011), determining what works optimally for whom and where (Rosen *et al.*, 2013).

From an operational perspective, three stress nodes were identified in the clients’ flow (Figs. 1 and 2). By assessing the RTE, stability and entropy on them, decision-makers can analyse how AOTs help to improve the technical performance of the MH ecosystem (García-Alonso *et al.*, 2019b, 2019a). Regarding the acute hospital care (R2) and non-acute time-limited hospital care services (R4–R6), potential removal of AOTs makes the MH system less efficient, decreases its stability (becomes more sensitive to small changes) and results in entropy (the variability in service offer decrease and the ecosystem becomes less efficient to match specific clients’ needs). In acute day hospital (D1) care, the RTE on average slightly decreased while the stability and entropy increased; this type of care is less sensitive to small changes and has to be more flexible to meet new clients’ needs.

After a potential AOT removal, the RTE assessment highlighted that if decision-makers want to reach a similar equilibrium to the original one, a significant investment should be made to increase the number of beds and workforces in residential hospital care and places in day care. Another solution would be the implementation of specific new services, such as compulsory treatment orders, case management (intensive or non-intensive), collaborative care or residential crisis programs, among others (Gaynes *et al.*, 2015). While other alternatives may exist for replacing the teams under study, MH care should prioritize being person-centred, holistic and carefully balanced between hospital and community care (Thornicroft and Tansella, 2014). Inadequately designed substitute interventions could lead to fragmentation and regression towards institutionalization.

## Strengths and limitations

The present study has the strength of estimating the effect of a complex type of care on a regional MH system by simulating its removal and, consequently, the alternative client’s flow into the ecosystem. It was achieved by implementing a causal model which helped the identification of stress nodes at the time that integrates the original rates with expert-based modified rates in an operational model to assess the impact of a specific type of care on the performance of the MH system.

However, several limitations must also be acknowledged. Clients and families did not participate in the design of the causal model. As the target population of the implemented intervention, service users should be included in the impact analysis process for a full overview of the ecosystem (Johnson *et al.*, 2022). Along the same lines, although the RTE assessment lets us understand the operational relevance of the AOTs, this type of care provides a holistic and personalized treatment to specific clients, increasing their satisfaction and autonomy. For this reason, technical performance assessment should include data about the quality of care (Almeda *et al.*, 2022b).

Further studies could benefit from the application of this methodology to more services and programmes in different MH systems. This could evolve in studies of comparative efficiency, comparing first with themselves and then with other regions or countries, determining what effects are similar in different contexts and what features of the intervention are more sensitive to the context at the local level.

## Conclusions

This research identified the AOTs as a non-acute low-intensity mobile care service rather than high-fidelity ACT teams. The development of the causal model provided a clear view of the key components and their relationships that would be affected by removing the AOTs. It combines micro-level information by understanding the clients’ flow according to the clinician and managers (nano-level) knowledge through time.

The causal model was then able to identify the stress node impacted by the AOTs (and their potential removal) in both the MH system of Bizkaia and within its context, including acute hospital care, non-acute time-limited hospital care and acute day hospital care services in the first and family and shelter/social services for the second.

Finally, the RTE analysis showed the negative impact of removal of the AOTs on the whole MH system and also each catchment area.

The methodology applied from an ecosystem perspective could help in the complex debate about how to balance the provision of the various components of MH systems and assist in providing greater specificity about where to invest according to an area’s attributes.

## Supporting information

Almeda et al. supplementary material 1 Almeda et al. supplementary material

Almeda et al. supplementary material 2 Almeda et al. supplementary material

Almeda et al. supplementary material 3 Almeda et al. supplementary material

## Data Availability

Data available in Supplementary Material 3.

## Supplementary material

The supplementary material for this article can be found at https://doi.org/10.1017/S2045796025000125.
