# Supplementary material for: Modelling the impact on a local mental health system of previously implemented care programs: the experience of assertive outreach teams in Bizkaia (Spain)
Source: Epidemiol Psychiatr Sci. 2025 Mar 17;34:e20. doi: 10.1017/S2045796025000125 (PMC11955424; doi:10.1017/S2045796025000125)
Supplement: Almeda et al. supplementary material 1 — Almeda et al. supplementary material [file S2045796025000125sup001.docx]

**Assertive Outreach Teams (AOTs) Description**

**AOTs in Bizkaia MH system**

Focusing our attention to AOTs, it is worthy to highlight the role of this service as an alternative care to hospitalizations. The AOTs in Bizkaia provides mobile outpatient care to people suffering from severe mental disorders. ACT teams are integrated in the Mental Health Network of Bizkaia, specifically in the community care and intermediate resources. Currently, in Bizkaia there are 5 ACT teams, which provide care to the following comarcas: Comarca Interior, Ezkerraldea, Uribe, Homeless Bilbao and Bilbao. The Homeless Bilbao ACT team provides care for homeless people diagnosed of severe mental disorder and its main objective is to integrate them in the MH network. ACT teams work in coordination with community mental health centres and hospitals.

**Associated Workforce**

Each AOT staff consists of a nurse, one auxiliary nurse, one part-time psychiatrist and one part-time social worker (Table 1).

Table 1. Workforce capacity in AOTs (35 hours per week) (Atlas Bizkaia, 2015)

| **Assertive Community Treatment (ACT)** | **Psychiatrist** | **Nurse** | **Auxiliar nurse** | **Social worker** | **Occupational therapist** | **Administrative**  **assistant** |
| --- | --- | --- | --- | --- | --- | --- |
| COMARCA INTERIOR ACT | 0.5 | 1 | 1 | 0.48 |  |  |
| EZKERRALDEA ACT | 0.5 | 1 | 1 | 0.48 | 1 |  |
| URIBE  ACT | 1 | 2 | 1 | 1 |  |  |
| HOMELESS BILBAO ACT | 0.7 | 0.5 | 0.5 |  |  |  |
| BILBAO  ACT | 1.3 | 1.8 | 2 | 0.5 |  |  |
| TOTAL | 5 | 8.3 | 7 | 3.36 | 0.2 | 1 |

Services are available from 9.30h to 16.30h. Users assigned to the assertiver outreach programme can contact the team by using a mobile phone.

**Users Inclusion Criteria**

The inclusion criteria for AOTs’ users are: 1) be aged between 18 and 65 years old, 2) suffering from a severe mental disorder (schizophrenia and affective disorders), 3) have a previous history of poor engagement to standard community mental health services and 4) having poor psychosocial functioning. Currently, each AOTs provide care for 30-40 service users.
