# Supplementary material for: Modelling the impact on a local mental health system of previously implemented care programs: the experience of assertive outreach teams in Bizkaia (Spain)
Source: Epidemiol Psychiatr Sci. 2025 Mar 17;34:e20. doi: 10.1017/S2045796025000125 (PMC11955424; doi:10.1017/S2045796025000125)
Supplement: Almeda et al. supplementary material 2 — Almeda et al. supplementary material [file S2045796025000125sup002.docx]

**Relative Technical Efficiency Analysis per Scenario and Catchment Area**

**Scenario 1: Intervention Impact on Acute Hospital Care (R2) Services Performance**

In S1 and, respectively, for the “low demand of care” and “high demand of care” situations, results showed a reduction of -0.97% (from 0.913 to 0.904) and -1.47% (from 0.913 to 0.9) on the global RTE on average. In the first situation (S1+“low demand of care”), Gernica (1.66%), Durango (1.55%) and Sestao (1.02%) MH areas increased their RTE on average while the rest of the areas decreased it. In the second (S1+“high demand of care”), all catchment areas decreased their RTE on average (Table 1S).

The statistical error was always lower than 2.5%, and the probability of having an RTE greater than 0.75 remained almost constant.

Globally, in S1 the system's stability decreased in both situations, up to -4.34% (from 60.34 to 57.73) and -5.14% (from 60.34 to 57.24) respectively. However, in Gernika (4.51%), Barakaldo (0.7%) and Durango (0.43%) the stability increased in the “low demand of care” situation.

In S1, the entropy decreased up to -2.18% (from 42.59 to 41.66) and -2.04% (from 42.59 to 41.72) respectively. Nevertheless, the entropy increased in Durango (11.29%), Gernika (11.09%), Derio (5.58%) and Ortxarkoaga (4.92%) in the “low demand of care” situation, as well as in Bermeo (7.24%) in the “high demand of care” situation. On the other hand, entropy decreased a lot in Santurzi (-7.41%) and Zalla (-7.09%) in the “low demand of care” situation and in Basauri (-13.88%), Etxaniz (-11.65%) and Erandio (-6.46%) in the “high demand of care” one.

Table 1S. Acute hospital care (R2) scenario (S1) results, variation (%) in brackets.

| **Areas** | **Relative technical efficiency (RTE) on average** | | | **Probability of having an RTE score greater than 0.75** | | | **RTE error** | | | **Stability of the ecosystem (%)** | | | **Shannon’s entropy (%)** | | |
| --- | --- | --- | --- | --- | --- | --- | --- | --- | --- | --- | --- | --- | --- | --- | --- |
|  | **Original** | **LDC** | **HDC** | **Original** | **LDC** | **HDC** | **Original** | **LDC** | **HDC** | **Original** | **LDC** | **HDC** | **Original** | **LDC** | **HDC** |
| **Global (Bizkaia MH ecosystem)** | 0.913 | 0.904  (-0.97) | 0.9  (-1.47) | 100 | 99.97  (-0.03) | 99.95  (-0.05) | 0.05 | 0.053  (6.53) | 0.09  (79.72) | 60.34 | 57.73  (-4.34) | 57.24  (-5.14) | 42.59 | 41.66  (-2.18) | 41.72  (-2.04) |
| **Ajuriagerra** | 0.915 | 0.908  (-0.77) | 0.913  (-0.22) | 100 | 100  (0) | 100  (0) | 0.131 | 0.278  (112.5) | 0.257  (96.53) | 63.71 | 63.62  (-0.14) | 63.74  (0.05) | 32.15 | 32.98  (2.58) | 32.21  (0.19) |
| **Barakaldo** | 0.889 | 0.9  (1.24) | 0.874  (-1.69) | 100 | 100  (0) | 100  (0) | 0.49 | 0.247  (-49.53) | 0.475  (-3) | 58.5 | 58.91  (0.7) | 58.13  (-0.63) | 46.25 | 46.78  (1.15) | 45.42  (-1.79) |
| **Basauri** | 0.92 | 0.904  (-1.74) | 0.9  (-2.17) | 100 | 100  (0) | 100  (0) | 0.289 | 0.384  (32.82) | 0.266  (-8) | 63.12 | 60.27  (-4.51) | 62.64  (-0.76) | 43.43 | 40.54  (-6.65) | 37.4  (-13.88) |
| **Bermeo** | 0.924 | 0.902  (-2.38) | 0.908  (-1.73) | 100 | 100  (0) | 100  (0) | 0.48 | 0.475  (-0.97) | 0.261  (-45.67) | 61.71 | 59.74  (-3.19) | 59.86  (-3) | 40.88 | 42.46  (3.86) | 43.84  (7.24) |
| **Derio** | 0.915 | 0.91  (-0.55) | 0.906  (-0.98) | 100 | 99.9  (-0.1) | 99.8  (-0.2) | 0.235 | 0.212  (-9.74) | 0.195  (-17.1) | 60.96 | 58.36  (-4.27) | 58.33  (-4.32) | 36.02 | 38.03  (5.58) | 37.41  (3.86) |
| **Durango** | 0.903 | 0.917  (1.55) | 0.899  (-0.44) | 100 | 100  (0) | 100  (0) | 0.204 | 0.378  (85.83) | 0.272  (33.81) | 62.8 | 63.07  (0.43) | 62.37  (-0.68) | 39.33 | 43.77  (11.29) | 39.51  (0.46) |
| **Erandio** | 0.924 | 0.907  (-1.84) | 0.91  (-1.52) | 100 | 99.9  (-0.1) | 99.7  (-0.3) | 0.286 | 0.344  (20.43) | 0.323  (13.23) | 61.14 | 58.37  (-4.53) | 58.53  (-4.27) | 37.94 | 36.91  (-2.71) | 35.49  (-6.46) |
| **Ercilla** | 0.923 | 0.907  (-1.73) | 0.914  (-0.98) | 100 | 100  (0) | 100  (0) | 0.232 | 0.097  (-58.23) | 0.275  (18.54) | 66.2 | 63.65  (-3.85) | 63.72  (-3.73) | 32.4 | 32.67  (0.83) | 31.27  (-3.49) |
| **Etxaniz** | 0.933 | 0.909  (-2.57) | 0.911  (-2.36) | 100 | 100  (0) | 100  (0) | 0.412 | 0.235  (-42.96) | 0.316  (-23.42) | 64.85 | 63.57  (-1.98) | 61.33  (-5.44) | 36.04 | 32.92  (-8.66) | 31.84  (-11.65) |
| **Galdakao** | 0.922 | 0.901  (-2.28) | 0.901  (-2.28) | 100 | 100  (0) | 100  (0) | 0.287 | 0.371  (28.97) | 0.484  (68.56) | 63.37 | 62.37  (-1.57) | 60.03  (-5.28) | 41.94 | 39.54  (-5.72) | 40.03  (-4.55) |
| **Gernika** | 0.904 | 0.919  (1.66) | 0.9  (-0.44) | 100 | 100  (0) | 100  (0) | 0.347 | 0.355  (2.47) | 0.206  (-40.72) | 60.52 | 63.25  (4.51) | 62.61  (3.45) | 38.52 | 42.79  (11.09) | 38.33  (-0.49) |
| **Ortuella** | 0.88 | 0.877  (-0.34) | 0.872  (-0.91) | 100 | 100  (0) | 100  (0) | 0.337 | 0.403  (19.61) | 0.458  (35.76) | 58.49 | 58.36  (-0.21) | 58.22  (-0.45) | 43.76 | 43.09  (-1.53) | 43.46  (-0.69) |
| **Ortxarkoaga** | 0.915 | 0.913  (-0.22) | 0.913  (-0.22) | 100 | 100  (0) | 100  (0) | 0.215 | 0.284  (32.26) | 0.27  (25.68) | 63.69 | 63.6  (-0.14) | 63.69  (0) | 32.31 | 33.9  (4.92) | 33  (2.14) |
| **Portugalete** | 0.916 | 0.909  (-0.76) | 0.909  (-0.76) | 100 | 100  (0) | 99.9  (-0.1) | 0.235 | 0.165  (-29.61) | 0.33  (40.44) | 61 | 60.79  (-0.36) | 58.44  (-4.21) | 36.07 | 37.13  (2.94) | 36.48  (1.14) |
| **Santurzi** | 0.932 | 0.91  (-2.36) | 0.909  (-2.47) | 100 | 99.7  (-0.3) | 100  (0) | 0.218 | 0.198  (-8.96) | 0.302  (38.88) | 63.72 | 58.48  (-8.22) | 60.72  (-4.71) | 38.48 | 35.63  (-7.41) | 36.86  (-4.21) |
| **Sestao** | 0.883 | 0.892  (1.02) | 0.875  (-0.91) | 100 | 100  (0) | 100  (0) | 0.364 | 0.331  (-8.86) | 0.31  (-14.61) | 58.42 | 58.52  (0.16) | 58.29  (-0.24) | 44.72 | 46.16  (3.22) | 44.11  (-1.36) |
| **Uribe** | 0.93 | 0.908  (-2.37) | 0.907  (-2.47) | 100 | 100  (0) | 99.7  (-0.3) | 0.258 | 0.289  (11.86) | 0.276  (6.98) | 63.98 | 60.72  (-5.1) | 58.53  (-8.52) | 37.21 | 36.92  (-0.78) | 35.76  (-3.9) |
| **Zalla** | 0.91 | 0.885  (-2.75) | 0.874  (-3.96) | 100 | 100  (0) | 100  (0) | 0.37 | 0.447  (20.72) | 0.541  (46.25) | 62.12 | 58.5  (-5.83) | 58.15  (-6.4) | 46.83 | 43.51  (-7.09) | 44.83  (-4.27) |

*Notes:* (LDC) “Low demand of care” situation; (HDC) “High demand of care” situation.

**Scenario 2: Intervention Impact on Non-acute Time-limited Hospital Care (R4-R7) Services Performance**

In scenario 2 (S2), the global RTE on average decreased slightly by -0.26 in the “low demand of care” situation (from 0.888 to 0.885), but it decreased by -1.04 in the “high demand of care” one (from 0.888 to 0.878). Ajuriagerra (6.17%) and Ortxarkoaga (6.17%) increased their RTE on average in the “low demand of care” situation, but they came back to their original scores in the “high demand of care” one (-0.47% and -0.12%). In Derio (respectively, -4.41% and -3.14%), Erandio (-4.04% and -2.73%) and Uribe (-3.61% and -2.52%) showed the greatest decrease of the RTE on average. The statistical error was always below 2.5% (Table 2S).

For the whole ecosystem, the probability of having an RTE greater than 0.75 remained almost constant (from 96.1% to 96.62%). Although in the “low demand of care” situation, Zalla showed a high decrease (-8.51%), followed by Gernika (-3.14%) and Durango (-1.93%). Nevertheless, in these catchment areas the probability gets closer to the initial situation in the “high demand of care” situation.

The global stability was almost constant (-0.62%) for the “low demand of care” situation but in the “high demand of care” one it decreased up to -4.93%. Ajuriagerra (4.92%) and Ortxarkoaga (4.57%) increased their stability, while Gernika (-9.74%), Derio (-6.95%), Erandio (-6.9%), Uribe (-6.63%), Ercilla (-3.69%) and Zalla (-2.57%) showed a significant decrease in the “low demand of care” situation. However, Ajuriagerra (0.23%) and Ortxarkoaga (-4.51%) decrease together with Derio (-7.27%), Erandio (-7.07%), Uribe (-6.97%), and Ercilla (-3.71%), while Gernika (0.07%) and Zalla (-0.1%) get closer to the initial situation in the "high demand of care" situation.

Finally, the entropy of the ecosystem remained almost constant in the “low demand of care” situation, but it decreased up to -1.33 in the “high demand of care” one. In the first situation, Derio significantly reduced its entropy (-10.37%), while Ortxarkoaga (12.76%), Zalla (11.59%) and Santurzi (5.6%) increased it. On the other hand, most of the catchment areas reduced their entropies in the “high demand of care” situation, except for Santurzi (1.82%) and Durango (1.31%).

Table 2S. Non-acute time-limited hospital care (R4-R7) scenario (S2) results, variation (%) in brackets.

| **Areas** | **Relative technical efficiency (RTE) on average** | | | **Probability of having an RTE score greater than 0.75** | | | **RTE error** | | | **Stability of the ecosystem (%)** | | | **Shannon’s entropy (%)** | | |
| --- | --- | --- | --- | --- | --- | --- | --- | --- | --- | --- | --- | --- | --- | --- | --- |
|  | **Original** | **LDC** | **HDC** | **Original** | **LDC** | **HDC** | **Original** | **LDC** | **HDC** | **Original** | **LDC** | **HDC** | **Original** | **LDC** | **HDC** |
| **Global (Bizkaia MH ecosystem)** | 0.888 | 0.885  (-0.26) | 0.878  (-1.04) | 96.84 | 96.1  (-0.76) | 96.62  (-0.23) | 0.064 | 0.17  (163.2) | 0.112  (73.41) | 52.4 | 52.08  (-0.62) | 49.82  (-4.93) | 51.94 | 51.98  (0.08) | 51.25  (-1.33) |
| **Ajuriagerra** | 0.859 | 0.912  (6.17) | 0.855  (-0.47) | 99.7 | 99.8  (0.1) | 99.5  (-0.2) | 0.271 | 0.226  (-16.69) | 0.383  (41.22) | 53.77 | 56.41  (4.92) | 53.65  (-0.23) | 34.06 | 35.48  (4.17) | 33.25  (-2.38) |
| **Barakaldo** | 0.919 | 0.918  (-0.11) | 0.915  (-0.44) | 100 | 100  (0) | 100  (0) | 0.266 | 0.234  (-11.97) | 0.24  (-9.76) | 63.74 | 63.74  (0) | 63.65  (-0.14) | 34.41 | 33.37  (-3.02) | 33.61  (-2.32) |
| **Basauri** | 0.857 | 0.854  (-0.35) | 0.85  (-0.82) | 93.4 | 92.7  (-0.75) | 93.6  (0.21) | 0.373 | 0.539  (44.35) | 0.465  (24.71) | 51.82 | 51.59  (-0.44) | 51.5  (-0.61) | 49.18 | 50.34  (2.36) | 49.36  (0.37) |
| **Bermeo** | 0.88 | 0.878  (-0.23) | 0.877  (-0.34) | 86 | 86.3  (0.35) | 85.9  (-0.12) | 0.781 | 0.581  (-25.65) | 0.585  (-25.16) | 53.86 | 54.04  (0.34) | 53.7  (-0.31) | 48.97 | 46.98  (-4.06) | 48.39  (-1.18) |
| **Derio** | 0.861 | 0.823  (-4.41) | 0.834  (-3.14) | 100 | 99.7  (-0.3) | 99.9  (-0.1) | 0.273 | 0.238  (-12.88) | 0.215  (-21.29) | 57.96 | 53.93  (-6.95) | 53.74  (-7.27) | 40.99 | 36.74  (-10.37) | 35.58  (-13.2) |
| **Durango** | 0.897 | 0.884  (-1.45) | 0.88  (-1.9) | 98.6 | 96.7  (-1.93) | 96.9  (-1.72) | 0.488 | 0.539  (10.39) | 0.802  (64.39) | 53.69 | 53.49  (-0.37) | 53.25  (-0.81) | 54.89 | 55.07  (0.33) | 55.61  (1.31) |
| **Erandio** | 0.915 | 0.878  (-4.04) | 0.89  (-2.73) | 100 | 100  (0) | 100  (0) | 0.196 | 0.284  (44.9) | 0.297  (51.2) | 62.69 | 58.36  (-6.9) | 58.25  (-7.07) | 43.94 | 43  (-2.14) | 42.89  (-2.39) |
| **Ercilla** | 0.919 | 0.922  (0.33) | 0.913  (-0.65) | 100 | 100  (0) | 100  (0) | 0.304 | 0.25  (-17.9) | 0.183  (-39.7) | 66.2 | 63.75  (-3.69) | 63.74  (-3.71) | 33.8 | 34.89  (3.22) | 32.69  (-3.28) |
| **Etxaniz** | 0.92 | 0.92  (0) | 0.913  (-0.76) | 100 | 100  (0) | 100  (0) | 0.208 | 0.173  (-16.74) | 0.179  (-14.27) | 63.74 | 63.66  (-0.12) | 63.62  (-0.19) | 35.41 | 36.5  (3.08) | 33.4  (-5.68) |
| **Galdakao** | 0.858 | 0.852  (-0.7) | 0.847  (-1.28) | 93.5 | 93.1  (-0.43) | 93.4  (-0.11) | 0.392 | 0.263  (-32.92) | 0.55  (40.29) | 51.92 | 51.47  (-0.88) | 51.47  (-0.88) | 49.42 | 50.74  (2.67) | 49.17  (-0.51) |
| **Gernika** | 0.895 | 0.889  (-0.67) | 0.889  (-0.67) | 82.7 | 80.1  (-3.14) | 81.8  (-1.09) | 0.584 | 0.582  (-0.4) | 0.643  (10.02) | 53.46 | 48.25  (-9.74) | 53.49  (0.07) | 50.16 | 49.81  (-0.7) | 48.79  (-2.73) |
| **Ortuella** | 0.92 | 0.918  (-0.22) | 0.913  (-0.76) | 100 | 100  (0) | 100  (0) | 0.225 | 0.235  (4.81) | 0.282  (25.78) | 63.72 | 63.71  (-0.02) | 63.77  (0.07) | 34.53 | 33.91  (-1.8) | 31.62  (-8.43) |
| **Ortxarkoaga** | 0.859 | 0.912  (6.17) | 0.858  (-0.12) | 99.9 | 99.7  (-0.2) | 99.2  (-0.7) | 0.293 | 0.334  (14.12) | 0.258  (-11.67) | 53.81 | 56.27  (4.57) | 51.39  (-4.51) | 34 | 38.34  (12.76) | 33.86  (-0.41) |
| **Portugalete** | 0.918 | 0.919  (0.11) | 0.913  (-0.54) | 100 | 100  (0) | 100  (0) | 0.212 | 0.213  (0.46) | 0.17  (-20.06) | 63.72 | 63.71  (-0.02) | 63.63  (-0.14) | 34.57 | 35.56  (2.86) | 33.31  (-3.64) |
| **Santurzi** | 0.892 | 0.888  (-0.45) | 0.889  (-0.34) | 100 | 100  (0) | 100  (0) | 0.121 | 0.163  (35.11) | 0.203  (68.47) | 62.82 | 62.22  (-0.94) | 62.34  (-0.76) | 31.27 | 33.02  (5.6) | 31.84  (1.82) |
| **Sestao** | 0.92 | 0.919  (-0.11) | 0.913  (-0.76) | 100 | 100  (0) | 100  (0) | 0.248 | 0.207  (-16.31) | 0.244  (-1.5) | 63.74 | 63.71  (-0.05) | 63.75  (0.02) | 34.97 | 34.79  (-0.51) | 32.63  (-6.69) |
| **Uribe** | 0.913 | 0.88  (-3.61) | 0.89  (-2.52) | 100 | 100  (0) | 100  (0) | 0.277 | 0.479  (73.15) | 0.49  (77.13) | 62.54 | 58.39  (-6.63) | 58.18  (-6.97) | 45 | 43.84  (-2.58) | 43.47  (-3.4) |
| **Zalla** | 0.773 | 0.768  (-0.65) | 0.772  (-0.13) | 89.3 | 81.7  (-8.51) | 89  (-0.34) | 0.181 | 0.135  (-25.48) | 0.124  (-31.42) | 52.27 | 50.93  (-2.57) | 52.22  (-0.1) | 20.45 | 22.82  (11.59) | 19.22  (-6.01) |

*Notes:* (LDC) “Low demand of care” situation; (HDC) “High demand of care” situation

**Scenario 3: Intervention Impact on Acute Health Day Care (D1) Services Performance**

In scenario 3 (S3), the RTE on average remained almost constant in both analysed situations. For the “low demand of care” one, the RTE on average decreased only -0.27%, while for the “high demand of care” was -0.37% for the whole ecosystem. None of the catchment areas increased its RTE on average (Table 3S).

The probability of having RTE greater than 0.75 also remained constant at all levels of analysis. The statistical error was always lower than 2.5%.

The stability of the global ecosystem increased up to 3.42% and 3.34% respectively for both situations. Bermeo decreased its stability up to -4.8%, while Zalla increased it up to 4.08% in the “high demand of care” situation.

The global entropy increased up to 0.94% in the “low demand of care” situation and up to 2.45% in the “high demand of care” one. Etxaniz (13.97%), Uribe (13.7%), Barakaldo (12.22%), Erandio (11.65%) and Sestao (11.01%) catchment areas increased the entropy significantly in the “high demand of care” situation, while Zalla decreased it up to -76.36% and -57.27% respectively.

Tabla 3S. Acute health day care (D1) scenario (S3) results, variation (%) in brackets.

| **Areas** | **Relative technical efficiency (RTE) on average** | | | **Probability of having an RTE score greater than 0.75** | | | **RTE error** | | | **Stability of the ecosystem (%)** | | | | **Shannon’s entropy (%)** | | |
| --- | --- | --- | --- | --- | --- | --- | --- | --- | --- | --- | --- | --- | --- | --- | --- | --- |
|  | **Original** | **LDC** | **HDC** | **Original** | **LDC** | **HDC** | **Original** | **LDC** | **HDC** | **Original** | **LDC** | **HDC** | **Original** | | **LDC** | **HDC** |
| **Global (Bizkaia MH ecosystem)** | 0.947 | 0.945  (-0.27) | 0.944  (-0.37) | 100 | 100  (0) | 100  (0) | 0.049 | 0.063  (28.28) | 0.113  (130.07) | 63.13 | 65.29  (3.42) | 65.23  (3.34) | 33.06 | | 33.37  (0.94) | 33.87  (2.45) |
| **Ajuriagerra** | NA | NA | NA | NA | NA | NA | NA | NA | NA | NA | NA | NA | NA | | NA | NA |
| **Barakaldo** | 0.963 | 0.963  (0) | 0.962  (-0.1) | 100 | 100  (0) | 100  (0) | 0.11 | 0.092  (-15.76) | 0.114  (4.15) | 73.45 | 71.94  (-2.06) | 71.94  (-2.06) | 15.3 | | 17.02  (11.24) | 17.17  (12.22) |
| **Basauri** | 0.939 | 0.934  (-0.53) | 0.932  (-0.75) | 100 | 100  (0) | 100  (0) | 0.206 | 0.27  (31.13) | 0.322  (56.61) | 64.78 | 64.13  (-1) | 63.71  (-1.66) | 38.37 | | 36.94  (-3.73) | 36.85  (-3.96) |
| **Bermeo** | 0.941 | 0.935  (-0.64) | 0.935  (-0.64) | 100 | 100  (0) | 100  (0) | 0.208 | 0.209  (0.37) | 0.303  (45.49) | 68.42 | 67.63  (-1.15) | 65.13  (-4.8) | 31.19 | | 31.43  (0.77) | 31.77  (1.86) |
| **Derio** | 0.963 | 0.962  (-0.1) | 0.961  (-0.21) | 100 | 100  (0) | 100  (0) | 0.101 | 0.098  (-2.82) | 0.124  (23.25) | 72.99 | 71.94  (-1.44) | 71.94  (-1.44) | 16.26 | | 16.89  (3.87) | 17.76  (9.23) |
| **Durango** | 0.939 | 0.932  (-0.75) | 0.931  (-0.85) | 100 | 100  (0) | 100  (0) | 0.339 | 0.317  (-6.46) | 0.172  (-49.32) | 64.99 | 63.87  (-1.73) | 63.56  (-2.21) | 37.41 | | 37.41  (0) | 36.93  (-1.28) |
| **Erandio** | 0.964 | 0.962  (-0.21) | 0.961  (-0.31) | 100 | 100  (0) | 100  (0) | 0.075 | 0.086  (13.78) | 0.069  (-8.44) | 73.49 | 71.94  (-2.11) | 71.94  (-2.11) | 15.97 | | 17.02  (6.57) | 17.83  (11.65) |
| **Ercilla** | 0.963 | 0.963  (0) | 0.961  (-0.21) | 100 | 100  (0) | 100  (0) | 0.082 | 0.09  (9.29) | 0.07  (-15.61) | 71.94 | 73.16  (1.69) | 71.94  (0) | 17.68 | | 16.65  (-5.83) | 18.82  (6.45) |
| **Etxaniz** | 0.964 | 0.963  (-0.1) | 0.961  (-0.31) | 100 | 100  (0) | 100  (0) | 0.125 | 0.074  (-40.83) | 0.117  (-5.83) | 73.8 | 71.94  (-2.51) | 71.94  (-2.51) | 15.03 | | 16.82  (11.91) | 17.13  (13.97) |
| **Galdakao** | 0.939 | 0.932  (-0.75) | 0.932  (-0.75) | 100 | 100  (0) | 100  (0) | 0.195 | 0.306  (56.43) | 0.238  (21.98) | 64.78 | 64.15  (-0.98) | 63.96  (-1.26) | 38.93 | | 35.85  (-7.91) | 36.34  (-6.65) |
| **Gernika** | 0.937 | 0.929  (-0.85) | 0.929  (-0.85) | 100 | 100  (0) | 100  (0) | 0.289 | 0.296  (2.33) | 0.209  (-27.65) | 64.26 | 63.68  (-0.91) | 63.71  (-0.86) | 37.32 | | 35.97  (-3.62) | 36.65  (-1.8) |
| **Ortuella** | NA | NA | NA | NA | NA | NA | NA | NA | NA | NA | NA | NA | NA | | NA | NA |
| **Ortxarkoaga** | 0.963 | 0.963  (0) | 0.962  (-0.1) | 100 | 100  (0) | 100  (0) | 0.111 | 0.113  (1.56) | 0.087  (-21.91) | 73.27 | 73.33  (0.08) | 71.94  (-1.81) | 16 | | 15.65  (-2.19) | 17.58  (9.87) |
| **Portugalete** | 0.964 | 0.963  (-0.1) | 0.962  (-0.21) | 100 | 100  (0) | 100  (0) | 0.102 | 0.106  (3.39) | 0.103  (0.04) | 73.4 | 73.54  (0.2) | 71.94  (-1.98) | 16.39 | | 15.04  (-8.24) | 17.54  (7.02) |
| **Santurzi** | 0.963 | 0.963  (0) | 0.961  (-0.21) | 100 | 100  (0) | 100  (0) | 0.076 | 0.109  (43.43) | 0.098  (28.22) | 73.33 | 73.05  (-0.38) | 71.94  (-1.89) | 16.25 | | 16.96  (4.37) | 16.99  (4.55) |
| **Sestao** | 0.964 | 0.961  (-0.31) | 0.962  (-0.21) | 100 | 100  (0) | 100  (0) | 0.107 | 0.059  (-45.11) | 0.07  (-34.77) | 73.31 | 71.94  (-1.86) | 71.94  (-1.86) | 16.44 | | 16.67  (1.4) | 18.25  (11.01) |
| **Uribe** | 0.963 | 0.962  (-0.1) | 0.961  (-0.21) | 100 | 100  (0) | 100  (0) | 0.096 | 0.071  (-25.65) | 0.061  (-36.47) | 73.23 | 71.94  (-1.76) | 71.94  (-1.76) | 16.21 | | 16.37  (0.99) | 18.43  (13.7) |
| **Zalla** | 0.83 | 0.827  (-0.36) | 0.828  (-0.24) | 100 | 100  (0) | 100  (0) | 0.066 | 0.087  (31.5) | 0.075  (13.47) | 58.67 | 61.06  (4.08) | 61.06  (4.08) | 1.1 | | 0.26  (-76.36) | 0.47  (-57.27) |

*Notes:* (LDC) “Low demand of care” situation; (HDC) “High demand of care” situation
